# The cross talk between gastric cancer stem cells and the immune microenvironment: a tumor-promoting factor

**DOI:** 10.1186/s13287-021-02562-9

**Published:** 2021-09-09

**Authors:** Jared Becerril-Rico, Eduardo Alvarado-Ortiz, Mariel E. Toledo-Guzmán, Rosana Pelayo, Elizabeth Ortiz-Sánchez

**Affiliations:** 1 Subdirección de Investigación Básica, Instituto Nacional de Cancerología, Secretaría de Salud, Ciudad de México, Mexico; 2 Programa de Posgrado en Ciencias Biológicas, Universidad Nacional Autónoma de México, Ciudad de México, Mexico; 3 Departamento de Bioquímica, Facultad de Medicina, Universidad Nacional Autónoma de México (UNAM), Ciudad de México, Mexico; 4 Centro de Investigación Biomédica de Oriente, Instituto Mexicano del Seguro Social, Delegación Puebla, Puebla, Mexico

**Keywords:** Cancer stem cells, Gastric cancer, Immunoregulation, Immunotherapy, Cancer microenvironment, Cell reprogramming, Cancer-associated immune cells

## Abstract

Cross talk between cancer cells and the immune system is determinant for cancer progression. Emerging evidence demonstrates that GC characteristics such as metastasis, treatment resistance, and disease recurrence are associated with a tumor subpopulation called gastric cancer stem cells (GCSCs). However, the specific interaction between GCSCs and the immune microenvironment is still under investigation. Although immune evasion has been well described for cancer stem cells (CSCs), recent studies show that GCSCs can also regulate the immune system and even benefit from it. This review will provide an overview of bidirectional interactions between CSCs and immune cells in GC, compiling relevant data about how CSCs can induce leukocyte reprogramming, resulting in pro-tumoral immune cells that orchestrate promotion of metastasis, chemoresistance, tumorigenicity, and even increase in number of cancer cells with stem properties. Some immune cells studied are tumor-associated macrophages (TAMs), neutrophils, Th17 and T regulatory (T_reg_) cells, mesenchymal stem cells (MSCs), and cancer-associated fibroblasts (CAFs), as well as the signaling pathways involved in these pro-tumoral activities. Conversely, although there are cytotoxic leukocytes that can potentially eliminate GCSCs, we describe mechanisms for immune evasion in GCSCs and their clinical implications. Furthermore, we describe current available immunotherapy targeting GCSC-related markers as possible treatment for GC, discussing how the CSC-modified immune microenvironment can mitigate or inactivate these immunotherapies, limiting their effectiveness. Finally, we summarize key concepts and relevant evidence to understand the cross talk between GCSCs and the immune microenvironment as an important process for effective design of therapies against GCSCs that improve the outcome of patients with GC.

## Introduction

Currently, non-communicable diseases particularly cancer stand out as the main cause of death in the world [1]. According to the World Health Organization International Agency for Research on Cancer, the estimated new cases and deaths from cancer worldwide in 2020 were 19.29 × 10^6^ and 9.96 × 10^6^, respectively, increasing mainly in low human development index countries [2]. Globally, gastric cancer (GC) ranks fifth in incidence among the major types of cancer, with 1,089,103 new cases per year, and represents the fourth leading cause of death from cancer (7.7%), with 768,793 deaths worldwide [2].

Among the causes of poor GC prognosis are tumor extent and grade, chemo- and radio-resistance, as well as its migration ability, which constitute some of the main challenges for current medicine [3].

Furthermore, despite research conducted the precise etiology of GC is still unknown. However, numerous risk factors for GC development have been acknowledged, the main risk factor being persistent infection of *Helicobacter pylori* [4].

In this sense, the origin of GC is a matter still under investigation, although there are theories formulated in this regard. In keeping with the hierarchical model of tumor evolution, a unique cancer stem cell (CSC) population, endowed with primitive stem cell properties and malignant characteristics, is most likely the foundation of an increasing number of malignant pathologies and the target of multiple investigations [5]. Notably, a strong relationship between CSCs and the surrounding immune microenvironment has been suggested to play a determinant role in cell fate decisions, including tumor initiation, progression, and metastasis [6, 7].

This review outlines the recent findings contributing to understanding the etiology of GC through the biology of developing gastric cancer stem cells (GCSCs) in the context of immune microenvironments. We hypothesize a strong interaction between GCSCs and immune cells, fostering reciprocal reprograming to promote cancer progression. In this sense, it is shown that GCSCs promote reprogramming of immune cells toward pro-tumoral phenotypes and that reprogrammed immune cells can increase the GCSC subset and their stemness capabilities, such as cell migration, clonogenicity, and treatment resistance, among other pro-tumoral activities. Furthermore, we discuss interesting mechanisms that could limit current immunotherapies against GC and specifically GCSCs. Taken together, by unraveling the mechanisms that regulate immune surveillance and responses in GCSCs, we may learn about new strategies to treat GC.

## GCSC and the hierarchical model of tumor progression: functional phenotypes and clinical significance

In the past, cancer was described as heterogenic mass of cells comprised by different subsets, and there have been various tumor models trying to explain this cellular heterogeneity. The hierarchical model of CSCs raises the existence of subsets of neoplastic cells with different proliferation and differentiation capacities, where those with the highest hierarchy and highest tumorigenic capacity are endowed with stem cell properties and different sequential differentiation potentials [8]. The CSC subset is characterized by showing self-renewal capacity, chemo and radio-resistance, as well as higher potential for metastasis through epithelial–mesenchymal transition (EMT), which make them stand out as highly relevant therapeutic targets [9, 10]. Many of these stem characteristics have been related to telomere length and telomerase activity. In this sense, it has been demonstrated that pancreatic CSCs show longer telomeres and higher telomerase activity than bulk tumor cells, which is related to the expression of pluripotency genes (Nanog, Sox2, Oct3/4). Furthermore, it was confirmed that telomerase inhibition results in pancreatic CSC apoptosis, making this a suitable therapy against CSCs, specifically in pancreatic cancer [11]. Importantly, although telomere shortening is associated with DNA instability and cell senescence [12], it is a process observed in cancer initiation which is then followed by telomere lengthening for chromosome stabilization and tumor progression [13].

According to the dynamic CSC model, a feedback loop can be established between CSCs and cancer progenitor cells, suggesting that cancer progenitor cells can also acquire stem characteristics under certain microenvironmental cues [14, 15]. In this regard, it is becoming clear that inflammatory conditions cooperate to provoke deregulations, mutations, cell fusion and others, ultimately leading CSC promoting conditions [16].

In gastric tissue, the origin of CSCs has been suggested to be from stem cells (SCs) [17, 18]. Under normal conditions, the stomach has two subsets of SCs found in gastric glands. The first is characterized by expressing CD44 and/or LGR5 markers and gives rise to differentiated cells of the gastric mucosa. The second SC is usually in a quiescent state but able to renew CD44^+^ or LGR5^+^ SCs and express Villin, TROY, and Mist1 markers [19]. Conversely, some studies propose bone marrow mesenchymal stem cells (BM-MSCs) as the cellular origin of GC. This theory is supported by findings where chronic inflammation caused by *Helicobacter* can promote attraction of BM-MSCs toward the damaged gastric epithelium, progressing these cells through metaplasia and dysplasia to gastric cancer [20]. In agreement with this theory, recent studies confirm that BM-MSCs promote gastric cancer progression, cell migration and tumorigenesis via c-Myc upregulation, a transcription factor involved in cell proliferation [21]. This mesenchymal stem cell (MSC) migration is dependent on chemotactic signals from GC, for example CXCL12 and TNF-α cytokines, a characteristic of MSC recently explored for cancer therapy to act as drug delivery tool or as an immune modulator [22]. Despite the aforementioned, MSCs have a dual role in many cancer types including GC, where MSCs can promote stemness, tumor growth, migration, and angiogenic activities, but have also been shown to induce inhibition of those tumorigenic activities; the role of this subset depends on the MSC model (amniotic, umbilical cord or bone marrow MSC origin) and the gastric cancer cell line used for experimental assays [23].

Consequently, CSC identification has become a relevant approach for cancer therapy; however, it is also a big challenge due to the low specificity of many markers for cancer cells. The most common markers described for GCSC identification are CD44 and the enzymatic activity of ADLH (aldehyde dehydrogenase) [24, 25]; however, additional biomarkers are still under study, such as CD24, CD326, LGR5, CD49f, CD54, CD90, CD71, CD133, as well as numerous transcription factors, including Sox2, Oct4, and Nanog [19, 26]. To identify GCSCs with higher phenotype specificity, researchers have tested some combinations of different previously described GCSC-related markers, such as CD44^+^CD54^+^ or CD44^+^CD24^+^ [27, 28]. In addition to their determination in gastric tissue, some studies have estimated GCSCs in peripheral blood samples and suggested that circulating GCSCs may initiate new tumor formation, as well as being associated with poor disease prognosis [27, 29].

Notably, the abundance of CD44^+^ and CD133^+^ cells and the expression of EpCAM, Oct4 and CD54 in GC have been correlated with TNM stage, tumor size, lymphovascular and distant metastases, poor prognosis and low survival [19].

Although the CSC theory shows interesting characteristics, it has several limitations or unclear points, such as when or from where do CSCs arise during carcinogenesis? What is their role in very early cancer stages? Is there really a hierarchical organization in the cancer model? Why are there so many reported GCSC phenotypes? Do all GCSC phenotypes have a common origin? Is there a specific function for each GCSC phenotype? As we will discuss, many of these limitations become evident when the interactions between GCSCs and immune cells are studied.

## The gastric cancer immune contexture

### Inflammation and microenvironment in the GC context

The study of tumor behavior, in vivo*,* is highly complex due to the presence of cellular and non-cellular components in the tumor microenvironment (TME). This involves various cell populations, extracellular matrix, hormones, growth factors, pathogens, among others, under a dynamic and multidirectional relationship between tumor cells, immune system, and microenvironment, which is decisive for establishment and tumor progression [30].

In this sense, the association between inflammation and cancer has been known for a long time [31]. In developed countries, nearly 23% of malignant diseases result from chronic inflammation produced by infectious agents, such as the hepatitis B and C virus in liver cancer, human papillomavirus in cervical and anogenital cancer, and *H. pylori* in stomach cancer [32]. Interestingly, *H. pylori* presence in gastric tissue has been associated with GCSC origin or maintenance. In this regard, one of the most studied mechanisms that show how *H. pylori* promotes GCSC is through its cytotoxin-associated gene A (CagA) protein, which in co-culture experiments has been demonstrated to be responsible for the increase of gastric cells with EMT and CSC properties, like CD44 expression, in a mechanism mediated by E-cadherin, NF-kB, and Zeb1/2 transcription factors [33]. Moreover, it has been revealed that *H. pylori* and the effects mediated by CagA promote loss of nuclear BRCA1 in gastric model cell lines, a protein with a critical role in protection against DNA double-strand breaks (DSB), thereby observing genomic instability; importantly, this fact could be related to *H. pylori*-associated carcinogenesis [34].

Despite these associations, not every inflammatory process favors the appearance of neoplasms, this depends on time and characteristics of the inflammatory process. However, in the context of a chronic inflammatory process, it has been suggested that persistent presence of inflammatory mediators could favor pro-oncogenic alterations [35].

There are different mechanisms to explain the association between chronic inflammation and carcinogenesis. For example, in cholangiocarcinoma it has been observed that exposure to pro-inflammatory cytokines IL-1β, TNF-α and INF-γ promotes nitric oxide release, and this correlates with the level of DNA damage [36]. Similarly, in gastric models it has been seen that the presence of *H. pylori* induces the production of hydrogen peroxide that results in DNA damage [37]. In this regard, in infection-associated cancer models nitrative and oxidative stress products have been found increased in cells with the presence of CSC-related markers (CD133 and Oct3/4) [38].

As discussed, the immune system plays a prominent role in cancer onset; however, it can also contribute to cancer progression. For example, pro-inflammatory cytokines such as TNF-α, IL-6, IL-1, and IL-17 can be anti-tumoral by stimulating pro-inflammatory and cytotoxic environments, but these cytokines can also have pro-tumoral activities due to their ability to stimulate signaling pathways associated with cell proliferation, survival, and angiogenesis [35]. Similarly, cytokines such as IL-1β, TGF-β1, and IL-6 can have pro-tumoral effects derived from their ability to stimulate cell migration, as well as secretion of metalloproteinases, pro-angiogenic factors like matrix metallopeptidase-9 (MMP-9), and vascular endothelial growth factor (VEGF) [39]. Thus, it has been suggested that the pro-tumor or anti-tumor activities of various mediators of the immune system depend on the intrinsic and extrinsic tumor cell conditions [40].

### Innate and adaptive immune system in feedback loop with GCSC

It is a fact that immune mediators are determinant factors for cancer development and progression; however, as will be discussed below, specific interaction between immune cells and GCSCs could be a major key for oncogenic process of GC.

Among the main leukocytes related to GCSCs are tumor-associated macrophages (TAMs), which correlate with prognosis in different types of cancer, including gastric cancer [41]. In GC, it is known that TAM-M2 predominance is associated with a worse prognosis due to anti-inflammatory activity, unlike TAM-M1, which generates a favorable prognosis due to a pro-inflammatory and anti-tumoral activity [42]. Furthermore, it has been observed that the infiltration of TAMs in gastric cancer is negatively correlated with the under-expression of CD3-zeta chain in T lymphocytes, which suggest that in gastric cancer TAMs could be another factor responsible for T cell activity [43].

TAMs have been involved in regulation of drug resistance and tumorigenicity by CSC. Downstream factors released by TAMs, especially milk-fat-globule-epidermal growth factor-VIII (MFG-E8) in cooperation with IL-6, activate STAT3 and Sonic Hedgehog signaling pathways, inducing chemoresistance in colon CSCs [44].

The presence of TAMs in GC has also been associated with promotion of metastasis, a process that appears to be regulated by GCSCs. In a co-culture study with monocytes and enriched GCSCs, overexpression of cytokines, monocyte chemoattractant protein-1 (MCP-1), IL-10, IFN-γ, and VEGF, was observed. Hence, pro-metastatic factors would be favoring migration of these GCSCs [43]. Although overexpression of these factors was evident in this study, it is necessary to demonstrate the stemness of this model.

It has also been shown, through co-culture studies between GC cells and TAM-M2, that M2 macrophages can promote the EMT process in GC. This is generated by a mechanism dependent on gastric cancer-derived mesenchymal stromal cells, which can secrete IL-6 and IL-8 to polarize TAMs toward the M2 phenotype, so that the polarized TAM-M2 can favor the EMT metastatic process in GC cells [45]. The importance of this fact lies in the relationship between the EMT process and stemness, since it has been confirmed that cancer cells subjected to EMT develop stemness markers such as high CD44 expression and increased ability to form tumor spheres [46]. Similarly, in a prostate model it was demonstrated that TAMs promote migration, EMT, and induce self-renewal of cancer cells through the release of the CCL5 chemokine, which activates the β-catenin/STAT3 signaling pathway to induce stemness [47].

Additionally, the presence of mesenchymal cells in the cancer microenvironment is important for CSC maintenance, mediated by TGF-β, a cytokine released by cancer-associated fibroblasts (CAFs) and MSCs, promoting stem characteristics in cancer cells [7].

It has also been studied, through in vitro and artificial assays, that MSCs fused with GC cells may generate cell hybrids with stem phenotype markers, in addition to increasing migration and proliferation capacity [48], a fact that supports to the possibility that MSCs may be involved in gastric carcinogenesis.

Exosome studies are an alternative approach for studying the role of MSC for the emergence of GCSCs. Several observations showed that BM-MSCs released exosomes containing ubiquitin protein ligase E3 component n-Recognin 2 (UBR2) could be internalized into GC cells and stimulate activation of the Wnt/β-catenin signaling pathway promoting cell migration, proliferation, and overexpression of stem-related genes [49].

**Fig. 1:**
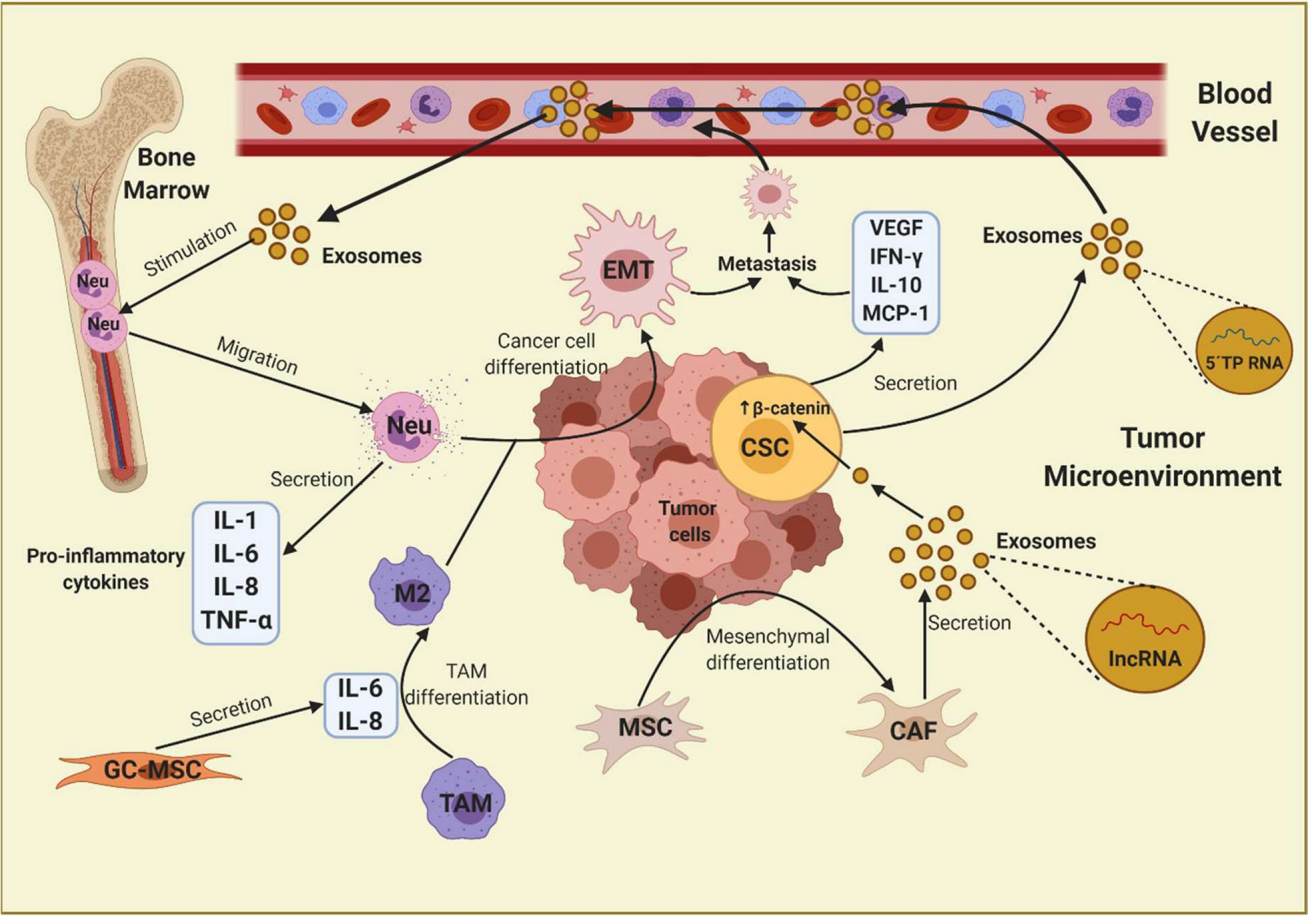
Antigen-presenting cells in gastric cancer immunoregulation. CSC, cancer stem cell; TAM, tumor-associated macrophage; M2, TAM type M2; Neu, neutrophil; MSC, mesenchymal stem cell; CAF, cancer-associated fibroblast; EMT, epithelial-mesenchymal transition; VEGF, vascular endothelial growth factor; MIP-2, macrophage inflammatory protein-2; lncRNA, long non-coding RNA; 5′TP RNA, 5′-Triphosphate RNA. TAM-M2 and neutrophils promote the EMT phenotype in GC. CSCs in turn recruit pro-inflammatory neutrophils from the bone marrow, through an exosome-mediated mechanism. Cancer cells stimulate differentiation of mesenchymal cells towards CAFs, which favor stemness characteristics and metastasis, through the action of non-coding RNA-containing exosomes

Following the paracrine regulation between CSCs and immune cells using exosomes, it is known that CAFs can favor enrichment of CSCs and promote metastasis, tumorigenicity, and chemoresistance through the release of exosomes containing H-19 non-coding RNA, whose activity stimulates pathways such as Wnt/β-catenin in CSCs. H19 seems to interact with miR-141, a β-catenin regulator, favoring β-catenin activity and overexpression [7]. Additionally, it was demonstrated that exosomes secreted by GC cells promote CAF differentiation from pericytes via PI3K/AKT and MEK/ERK cell pathway activation [50]. Completing this interaction loop between CSCs-CAFs, it has been reported that breast CSCs can secrete Hedgehog ligand (SHH), stimulating the Sonic Hedgehog signaling pathway in CAFs. Hedgehog signaling in CAFs promotes release of many ligands and growth factors such as fibroblast growth factor-7 and versican, which positively affect CSCs, increasing expansion and self-renewal [51]. These relevant interactions between CSCs, CAFs, and TAMs are outlined in Fig. 1.

Furthermore, in murine models, CSCs have been found to recruit and induce pro-tumor phenotypes in neutrophils through the release of RNA contained in exosomes, which travel to the bone marrow interacting with neutrophils, promoting their survival, proliferation, and IL-1β synthesis. CSCs release chemokines like CXCL1 and CXCL2 to recruit these pro-tumorigenic neutrophils, resulting in higher tumorigenicity and decreased survival in mice [52]. This suggests that CSCs recruit neutrophils from the bone marrow to benefit from their pro-inflammatory capacity, favoring the EMT process, with the subsequent generation of metastatic sites (Fig. 1). Association between tumor-associated neutrophils (TANs) and metastases is apparently caused by the release of IL-1β, IL-6, IL-8, IL-17, and TNF-α, which induce the EMT process in gastric cancer cells by activation of cell signaling pathways such as JAK2/STAT3 and ERK1/2 [53].

**Fig. 2:**
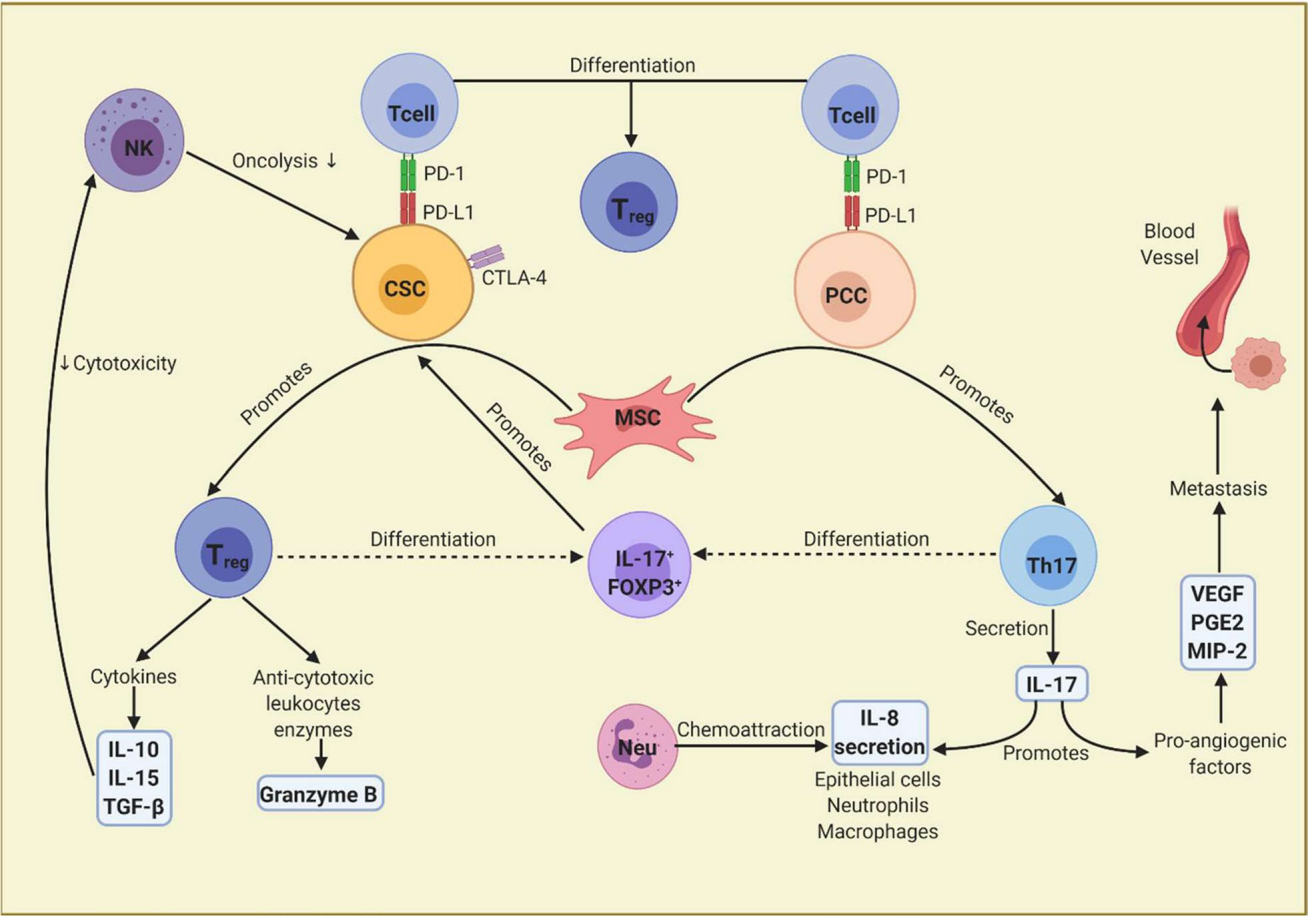
Lymphocytes and immune checkpoints in the gastric cancer immunoregulation. CSC, cancer stem cell; PCC, progenitor cancer cell; MSC, mesenchymal stem cell; Neu, neutrophil; NK, natural killer lymphocyte; VEGF, vascular endothelial growth factor; PGE2, prostaglandin E2; MIP-2, macrophage inflammatory protein-2. The interaction between MSCs and PCCs promotes lymphocyte differentiation towards the Th17 phenotype. Furthermore, interaction between MSCs and CSCs favors a T_reg_ phenotype. Differentiation of both, T_reg_ and Th17 lymphocytes, into an intermediate IL-17^+^FOXP3^+^ phenotype is possible, and the presence of this population has been shown to promote stemness in cancer cells. Expression of immune checkpoints can favor immune evasion in tumors, however, it can also stimulate stemness characteristics

Regarding the lymphocyte compartment, the Th17 phenotype is the most abundant type of lymphocyte within GC tumor tissue and it is associated with lower survival in GC patients. The interleukin produced by Th17 lymphocytes, IL-17, has been found to be elevated in tumor tissue and peripheral blood of GC patients, specifically in those with metastases [54]. Although Th17 lymphocytes are usually viewed as a pro-inflammatory and totally opposite to anti-inflammatory regulatory T (T_reg_) cells, there is evidence that mixed phenotypes can exist between these cells, and these are associated with CSC induction. For example, it is known that T_reg_ IL-17^−^ lymphocytes can become IL-17^+^, transforming into a pro-inflammatory phenotype [55], while Th17 lymphocytes without transcription factor, FOXP3, expression can become to FOXP3^+^, functioning as T_reg_ lymphocytes [56]. These IL-17^+^FOXP3^+^ lymphocytes have been found in colorectal tumor tissue and have been shown to promote generation of CSCs in hypoxic environments by inducing activation of MAPK and AKT kinases in cancer cells (Fig. 2). Interestingly, this study demonstrates that in co-culture, IL-17^+^FOXP3^+^ lymphocytes induce stem characteristics in bone marrow sphere cells [57], a fact that supports the theory suggesting CSCs are bone marrow-derived, as theorized in a GC model [21].

Furthermore, regulation of Th17/T_reg_ plasticity or relationship seems to be dependent on interaction between MSCs, CSCs and progenitor cancer cells (PCCs). The interaction between MSCs and CSCs presumably generates the release of MSC-derived TGF-β that induces differentiation of lymphocytes toward T_reg_ phenotypes. However, MSCs-PCCs induce lymphocyte differentiation toward a Th17 phenotype (Fig. 2) [6]. In this way, GCSCs could be regulators of the Th17/T_reg_ ratio, whose imbalance has been associated with the development and progression of GC [58].

### Immune evasion in GCSCs

As mentioned, there are mechanisms in GC that could maintain an anti-inflammatory microenvironment through increase in T_reg_ and TAM-M2 populations; however, it is necessary to explain specific mechanisms of immune evasion used by GCSCs to avoid the activities of anti-tumoral leukocytes. Studying these mechanisms will help to identify challenges for treatments targeting GCSCs.

CD8^+^ T lymphocytes are a population whose presence in tumor tissue is a good prognostic factor in GC [59]. Moreover, CSCs evade CD8^+^ lymphocytes by down-regulating MHC-I (major histocompatibility complex type I), a process that significantly decreases the CSC susceptibility to the lytic activity of these lymphocytes, since antigen recognition is avoided. However, although MHC-I down-regulation allows CSCs to evade CD8 + lymphocytes, it makes them more susceptible to be attacked by natural killer (NK) lymphocytes, specialists in the recognition of cells with low levels of MHC-I [60].

Interestingly, in patients with GC, low cytotoxic activity of NK lymphocytes has been reported, as well as decreased cell numbers in the tumor microenvironment, a fact related to a high degree of tumor invasion, TNM stage, metastasis to lymph nodes, and shorter survival. Low NK cytotoxicity in gastric cancer patients has been described in microenvironments with increased presence of IL-10, TGF-β, as well as type M2 TAMs (Fig. 2) [61]. As mentioned, co-cultures of enriched GCSCs and TAMs generate overexpression of anti-inflammatory cytokines, so it is possible that these serve as mechanisms of GCSCs for immune evasion against NK cells. Despite the above, CSCs have been observed to be more susceptible to identification by NK lymphocytes, due to the overexpression of ligands such as MICA/B, Fas, DR5, NKp30, and NKp44 [60]. Therefore, NK cell studies may lead to interesting therapeutic approaches.

Like NK lymphocytes, dendritic cells (DCs) show a decreased cytotoxicity in tumor microenvironments and blood [62]. Despite the impairment in DC cytotoxicity, there is evidence that stimulating them with CSC CD44^+^ lysates generates an anti-tumor phenotype of dendritic cells, which in turn stimulates the cytotoxic activity of lymphocytes, decreasing tumor size and increasing survival in murine models [63]. An opposite effect has been noted when stimulating DCs with CSC CD44^−^CD133^+^ lysates, observing a defect in DC activation [64]. This fact demonstrates that the effect of CSCs on immune system regulation is dependent on the CSC phenotype, that is, not all CSC phenotypes suppress or stimulate the immune response.

Finally, immune checkpoints such as programmed death-ligand 1 (PD-L1) seem to have an immunological role in GCSCs. PD-L1 is a surface receptor that induces anergy in T cells through its interaction with PD-1, and is commonly expressed in different types of cancer, including GC, being related to immune evasion [41]. Different investigations have shown that PD-L1 expression is not equal in all cancer cell groups, for example, in head and neck, lung, and breast cancer PD-L1 expression is mainly associated with CD44^+^ cells, a marker closely associated with GCSCs [65]. PD-L1 expression evaluated in GCSCs has been associated with higher cell proliferation [66]. Other studies on PD-L1 in CSCs have shown that PD-L1 knockdown generates loss of stemness characteristics and reduced chemoresistance in CSCs; however, overexpressed PD-L1 favors tumorigenicity, chemoresistance, and production of CSC-associated proteins, like ALDH [67]. In this sense, expression of PD-L1 in GCSCs could provide them with the ability to evade the immune system, as well as to maintain stemness characteristics (Fig. 2).

### Stemness-related signaling pathways favored by immune activity

As previously described, the immune microenvironment of GC is composed by cellular and non-cellular components that together allow tumor progression. Many actions generated by those components are through stimulation of specific signaling pathways that, as reviewed throughout this manuscript, can be related to stemness in gastric cancer cells [68].

**Fig. 3:**
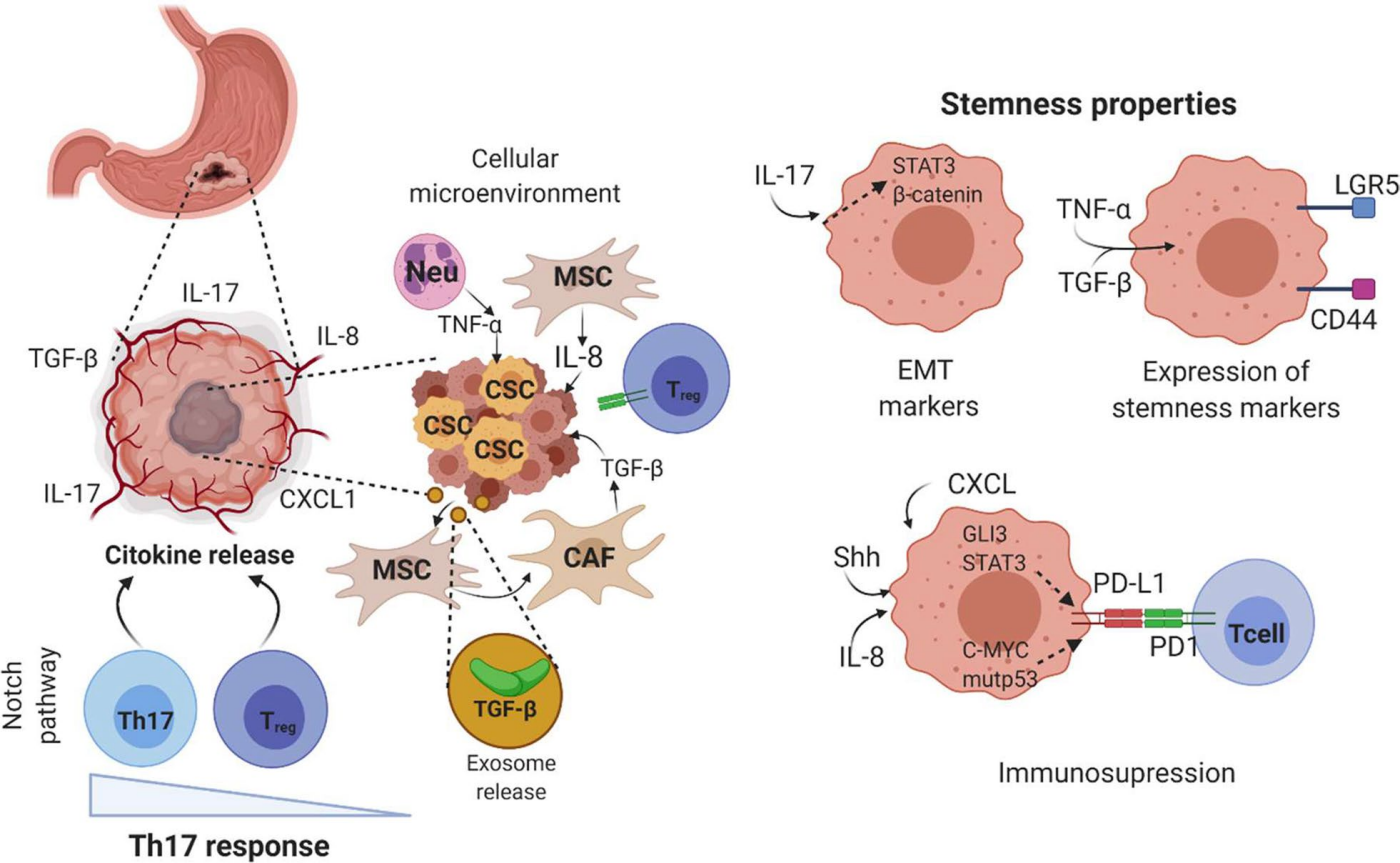
Stemness-related signaling pathways stimulated by the immune system. Neu, neutrophil; MSC, mesenchymal stem cell; CSC, cancer stem cell; EMT, epithelial mesenchymal transition; T_reg_, T regulatory lymphocyte; Shh, Sonic hedgehog. The Notch pathway regulates the balance between Th17 and Treg. The immune microenvironment regulates tumoral process such as EMT and expression of stemness markers, as well as immune evasion

Previously, it was mentioned that a corrupted balance between Th17/T_reg_ lymphocytes in GC shows an infiltrate with higher proportion of Th17 lymphocytes in the tumor microenvironment [58]. In this regard, it has been shown that the Notch pathway can function as a regulator of Th17 and T_reg_, a fact supported by an observed reduction in cytokine release from Th17- and FOXP3-expressing T_reg_ cells after Notch pathway blockade, without affecting Th17 or T_reg_ cellular proportions, suggesting that this signaling pathway is an important element to regulate the effect of the immune system over gastric cancer cells (Fig. 3) [69].

Pro-tumorigenic effects generated by Th17 cells are derived from IL-17β activity [54]. IL-17β seems to induce metastasis and tumorigenicity by overexpression of EMT markers and activation of AKT/GSK3-β/β-catenin pathways in cancer cells, resulting in expression of stemness markers such as Oct4, Sox2 and Nanog (Fig. 3) [70]. Other reports support that IL-17β can increase the migratory capacities of quiescent CSC (CD26^−^CXCR4^−^) through activation of the STAT3 pathway, suggesting that the effects of this cytokine are not limited to a particular signaling pathway [71]. However, the actions of IL-17 are not only over cancer cells, since this cytokine can also increase the expansion of mesenchymal stem cells in tumors, which, as previously discussed, can regulate the Th17/T_reg_ balance [70].

Throughout this work, we have discussed the role of TGF-β in cancer development and CSC induction; however, the mechanisms through which this is achieved have not been described. In this sense, the expression of GCSC-associated markers and stemness characteristics are apparently stimulated by the TGF-β/Smad pathway [72]. Other reports sustain that T_reg_-released TGF-β promotes the overexpression of LGR5 via activation of the canonical Wnt pathway, a process associated with poor prognosis in GC patients [73].

In addition, TNFα has been referred to as an inflammatory factor related to GC development. Demonstrating a close relationship with CSCs, TNFα ^(−/−)^ knockout mice did not express CSC markers, such as CD44, which establishes that great part of the stemness behavior may be related to the microenvironment generated by this cytokine, via a mechanism by which tumor necrosis factor receptor 1 (TNFR1) up-regulates Noxo1 and Gna14 pathways (Fig. 3) [74].

Finally, PD-L1 is also another immune component that promotes stemness though different signaling pathways. Recent studies note that PD-L1 is not only a receptor with immune functions, since there is evidence in xenograft models that it is a promoter of stemness characteristics in cancer, such as self-renewal, tumorigenesis, and chemoresistance, derived from interactions between PD-L1 and HMGA1, a transcription regulator protein that promotes the activation of PI3K/AKT and MAPK signaling pathways [67].

Promising results have been obtained using PD-L1 and PD-1 inhibitors. In a phase III trial with advanced GC patients evaluating the use of nivolumab, an IgG4 monoclonal antibody with a co-inhibitory signal on PD-1/PD-L1, results showed a 12-month overall survival of 26.6% compared with 10.9% in the placebo group, which shows a high number of patients without response to treatment [75]. Recently, it was described that it is possible to predict treatment response to PD-1 blockade in patients with gastric cancer, by studying chromatin accessibility of circulating CD8^+^ T cells, reporting better survival in patients with high chromatin openness at specific genomic positions in circulating CD8^+^ cells [76].

## Targeting GCSCs through the immune system

### Immune system as a tool in the treatment against GCSC

Currently, many therapies targeting CSC are being developed, including different inhibitors for signaling pathways or enzymes associated with stemness such as STAT3, Nanog, Sonic Hedgehog, Notch, mTORC1, ALDH, telomerase inhibitors, mimetics of non-coding RNAs like miR-34a, among other therapies [11, 77]. Limitations of these and other cancer treatments are due to low of specificity, since the mentioned therapies against CSCs can affect normal stem cells, generating problems in tissue renewal. For example, since LGR5 is a marker related to normal stem cells in gastric tissue and GCSCs, new therapies have been developed for eliminate LGR5^+^ cells in gastric cancer. Importantly, mTORC1 maintains self-renewal of LGR5^+^ populations, preventing cell differentiation, resulting in gastric tumorigenesis. These carcinogenic functions make LGR5 a potential therapeutic target; however, the use of mTORC1 inhibitors may generate antral gland atrophy due to poor tissue turnover, limiting its use for therapy [78].

As mentioned before, GCSCs interact with immune cells in the tumor microenvironment in order to avoid being eliminated by those immune cells. However, the immune system can be also a tool for gastric cancer treatment, though current immune therapy strategies targeting CSC markers. Table 1 shows examples of immune therapies with potential use against GCSCs.

**Table 1:** Immunotherapies targeting CSC markers with potential use against GCSC

Therapy	Target	Type of cancer	Type of study	Results	References
Antibody–drug conjugate	LGR5	Colon	Preclinical murine xenograft	Tumor stasis or regression in vivo Does not target healthy epithelia ↑Survival	[79]
Antibody RG7356	CD44	CD44^+^ solid tumors	Clinical phase I NCT01358903	Fever, headache and fatigue 21% of the patients presented disease stabilization No activation of macrophages Possible migration of monocytes to tumor tissue	[80]
DC-vaccination	Pool CSC antigens	Ehrlich carcinoma	Preclinical murine xenograft	↓ Tumor growth ↓ MDR and Bcl-2 ↑ Sensitivity to chemotherapy	[81]
DC-vaccination	Pool CSC antigens	Melanoma Squamous cell carcinoma	Preclinical syngeneic murine model	↓ Tumor growth ↓ Metastasis ↓ CSC features ↑ Survival	[82]
DC-vaccination	Pool CSC antigens	Breast	Preclinical cell lines	↑ Apoptosis ↑ IFN-γ	[83]
Immune checkpoint inhibition	PD-1	Melanoma	Clinical phase Ib NCT01704287	↑ Progression-free survival	[84]
Immune checkpoint inhibition	CTLA-4 PD-1	Colorectal	Preclinical syngeneic murine model	↑ CD8 + T cells	[85]
NK-activated cells	ALDH CD24 CD44 CD133	Breast	Preclinical cell lines	↓ CSCs populations	[86]
CAR-T	CD44-v6	AML MM	Preclinical murine xenograft	↑ Anti-tumor activity Specifically killed cancer cells ↑ IL-7/IL-15 efficacy	[87]
CAR-T	CD133	Glioblastoma	Preclinical patient-derived cells Preclinical murine xenograft	↑ CD133^+^cells elimination ↑ CD57 marker in lymphocytes ↑ Survival	[88]
CAR-T combined with Paclitaxel	CD54	Gastric	Preclinical murine xenograft	↑ Survival ↑ Anti-tumor activity ↓ Tumor growth	[89]

HA, hyaluronic acid; CSC, cancer stem cell; DC, dendritic cell; NK, natural killer; AML, acute myeloid leukemia; MM, multiple myeloma

Monoclonal antibodies targeting cell surface markers of CSCs show great potential to eliminate this subpopulation. An example is the recombinant monoclonal antibody RG7356 that blocks the binding of all CD44 isoforms to hyaluronic acid to reduce tumor growth in vivo by successfully phagocytizing CD44 + CSCs. However, in a phase I clinical trial involving sixty-five patients with different solid tumors, the efficacy of the antibody was moderate [80].

Conversely, immunotherapy based on cellular components provides a treatment with the most dynamic mechanism of action. For example, dendritic cell-based vaccination is a therapy based on the role of DCs as antigen-presenting cells (APCs) to CD8^+^ and CD4^+^ lymphocytes. The participation of DCs consists in obtaining the cells from patients and pulse them with tumor-associated antigens or tumor cell lysates in order to generate mature DCs able to induce cytotoxic T lymphocytes against tumor cells and CSCs [82]. This therapy has been demonstrated to induce tumor regression, increase apoptosis, and reduce metastasis in various types of cancer, such as melanoma, breast cancer, and colon [81–83].

Lymphocytes are another type of cells widely used in anti-CSC therapies. NK lymphocytes are innate immune system effector cells that do not need a previous encounter with an antigen to fulfill its cytotoxic function, an advantage in the treatment against cells that fail to express MHC efficiently, such as cancer cells and CSCs [60]. Therapy based on effector NK cells with assays carried out in pancreatic, breast, glioblastoma and sarcoma cell lines confirms elimination of those cell populations in a mechanism derived from upregulation of NK activation ligands MICA/B in CSC, and NKG2D receptors in NK cells, which generates the suppression of tumor growth [86].

Finally, immunotherapy using engineered T cells that express chimeric antigen receptors (CARs) is a therapy that contemplates the benefits of cellular and antibody-based treatments. Chimeric antigen receptor T cell (CAR-T) therapy consists in introducing autologous CD4^+^ and CD8^+^ lymphocytes modified to express CAR on their cellular surface, allowing them to recognize and respond to a specific antigen without a previous antigen-presenting process [90]. There is research using CAR-Ts that target markers such as CD44v6, CD133, EpCAM, and CD54, among others, resulting in efficient CSC elimination [87–89]. Combinatory therapy has also been used in a gastric cancer model, for example, combinatory treatment with CAR-Ts targeting CD54 in combination with paclitaxel chemotherapy or local stimulation with IL-12 generates longer survival and less tumor growth in xenograft models compared with monotherapy [89].

### Immunotherapy resistance related to GCSC microenvironment

It is evident that immunotherapy has promising results in plenty of preclinical studies; however, in clinical studies, immunotherapies face great limitations, such as treatment resistance [91].

Immunotherapy resistance is caused by many factors, among which the TME stands out. Thus, effects of stromal cells and other immunosuppressive cells, as well as metabolism-driven effects on TME facilitating conditions for cell infiltration, survival, and proliferation, among others, must be considered in order to develop immunotherapeutic strategies that could be applicable to patients [92].

For example, the presence of PD-L1 in the tumor microenvironment is a limiting factor in lymphocyte anti-cancer activity, therefore, considering the presence of PD-L1 is important for use of lymphocyte-based immunotherapies such as CAR-Ts [93].

Another possible cause of failure in CAR-T therapy is T cell exhaustion. This process generates progressive loss of function in lymphocytes due to an unfavorable state in the immune microenvironment. For example, high levels of IL-10, IL-35, TGF-β, as well as T_reg_ lymphocytes, have been shown to facilitate T cell exhaustion, promoting an immunosuppressive environment that is a great barrier for CAR-T therapy [93].

Like PD-1, high levels of the checkpoint receptor TIM-3 have been observed to result in failure of CAR-T treatment [94]. In this regard, it should be noted that in GC there is an elevated presence of the Gal-9 receptor, ligand of the TIM-3 lymphocyte receptor, whose axis generates the inactivation of lymphocyte [95].

Additionally, cellular components of the TME can contribute to anti-cancer treatment failure. It has been found that a high density of CAFs and TAM-M2 restricts the infiltration capacity of cytotoxic lymphocytes, probably due to the release of anti-inflammatory cytokines such as TGF-β and IL-10, respectively [96]. Similarly, exposure of NK lymphocytes to IL-10 and TGF-β affects their cytotoxic function [61].

In this sense, TME characteristics are important factors for immunotherapies based on NK, CD8^+^, and CAR-T lymphocytes and possibly on monoclonal antibodies.

Interestingly, cytotoxic activity of lymphocytes has been found to promote dedifferentiation of neoplastic cells, generating resistance to immunotherapy. In a study with melanoma patients using adaptive T cell transfer (ACT) therapy with MART1 antigen, a specific antigen in malignant melanoma, it was observed that patients resistant to immunotherapy generated dedifferentiation of tumor cells, which showed loss of the MART1 marker, a process derived after cytotoxic lymphocyte infiltration, and which appears to be dependent on TNF-α release in the tumor microenvironment [97]. This leads to the conclusion that a pro-inflammatory TME is not entirely beneficial for immunotherapies based on lymphocytes or monoclonal antibodies, at least in melanoma, but it is necessary to study the presence of this kind of therapy resistance in other cancer models.

As mentioned previously, in GC there are CAF, TAM-M2, T_reg_ lymphocytes, and Th17 lymphocytes present, which favor the synthesis of cytokines such as IL-10, IL-15, TGF-β, and IL-17 [41, 58]. The presence of these immune cells and cytokines is largely regulated by GCSCs, through various mechanisms already discussed [6]. In addition, the expression of immunological checkpoints such as Gal-9 and PD-L1 is maintained in GC, and the latter is also expressed in GCSCs [66]. Due to the above, the GC immune microenvironment represents a determining factor for resistance against immunotherapies, especially those focused on GCSCs.

Regarding an improvement in the effectiveness of anti-tumoral therapy, evidence suggests that it is not beneficial to focus therapies only toward the elimination of certain cellular subsets, or to affect a single relevant signaling pathway. Cancer is a complex disease that needs to be attacked from several sides. Drug combination is a reliable strategy that aims to eliminate cancer cells and prevent execution of pro-survival responses, yet it is equally important that such drugs be targeted based on the regulation of the immune TME, in order to avoid different mechanisms of resistance to immunotherapy, as well as favoring elimination of cancer cells, and controlling all the factors in the TME that malignant cells can take advantage of.

## Conclusion

CSCs represent an important cellular subset with outstanding functions in tumor progression, being related in GC with worse clinical outcome in patients. Herein, different mechanisms by which the immune microenvironment can be a contributing factor to carcinogenesis and GCSC induction were discussed, notably their role in the maintenance of stemness, metastasis, and development of immunoresistance and chemoresistance in GCSCs. This is a bidirectional interaction, since CSCs can stimulate, recruit, or differentiate leukocytes to favorable phenotypes or activities. The influence that CSCs exert over leukocytes is dependent of the specific CSC phenotype, due to the different expression of cytokines and other factors, such as RNA contained in exosomes and immunological checkpoints. Considering the existence of various GCSC phenotypes, it is necessary to investigate the specific function of each phenotype in the tumor microenvironment. Furthermore, as was discussed, the immune microenvironment promoted by GCSCs is a factor that alters the effectiveness of immunotherapies such as CAR-Ts and monoclonal antibodies, although this could be related to classic tumor therapy resistance. Therefore, the mentioned interaction between GCSCs and the immune system is a challenge for the treatment of GC, and an important factor to consider in the development of future strategies against cancer.

## Data Availability

Not applicable.

## Abbreviations

ACT: Adaptive T cell transfer
ALDH: Aldehyde dehydrogenase
BM-MSCs: Bone marrow mesenchymal stem cells
CAFs: Cancer-associated fibroblasts
CagA: Cytotoxin-associated gene A
CAR: Chimeric antigen receptor
CAR-Ts: Chimeric antigen receptor T cells
CSCs: Cancer stem cells
DCs: Dendritic cells
EMT: Epithelial–mesenchymal transition
GC: Gastric cancer
GCSCs: Gastric cancer stem cells
MCP-1: Monocyte chemoattractant protein-1
MHC-I: Major histocompatibility complex type I
MMP-9: Matrix metallopeptidase-9
MSCs: Mesenchymal stem cells
NK: Natural killer
PCCs: Progenitor cancer cells
PD-L1: Programmed death-ligand 1
SCs: Stem cells
TAMs: Tumor-associated macrophages
TANs: Tumor associated neutrophils
TME: Tumor microenvironment
TNFR1: Tumor necrosis factor receptor 1
T_reg_: Regulatory T
VEGF: Vascular endothelial growth factor
